# The impact of the UK ‘Act FAST’ stroke awareness campaign: content analysis of patients, witness and primary care clinicians’ perceptions

**DOI:** 10.1186/1471-2458-13-915

**Published:** 2013-10-02

**Authors:** Stephan U Dombrowski, Joan E Mackintosh, Falko F Sniehotta, Vera Araujo-Soares, Helen Rodgers, Richard G Thomson, Madeleine J Murtagh, Gary A Ford, Martin P Eccles, Martin White

**Affiliations:** 1 Institute of Health & Society, Newcastle University, Baddiley-Clark Building, Newcastle upon Tyne NE2 4AX, UK; 2 Division of Psychology, School of Natural Sciences, University of Stirling, Cottrell Building, Stirling FK9 4LA, UK; 3 Fuse, UKCRC Centre for Translational Research in Public Health, Newcastle University, Newcastle upon Tyne, UK; 4 Institute for Ageing & Health, Newcastle University, Newcastle upon Tyne NE2 4AE, UK; 5 Department of Health Sciences, University of Leicester, University Road, Leicester LE1 7RH, UK; 6 Acute Stroke Unit, Royal Victoria Infirmary, Newcastle upon Tyne Hospitals NHS Foundation Trust, Newcastle upon Tyne NE1 4LP, UK

**Keywords:** Delay, Stroke, Awareness, Mass-media campaign

## Abstract

**Background:**

The English mass media campaign ‘Act FAST’ aimed to raise stroke awareness and the need to call emergency services at the onset of suspected stroke. We examined the perceived impact and views of the campaign in target populations to identify potential ways to optimise mass-media interventions for stroke.

**Methods:**

Analysis of semi-structured interviews conducted as part of two qualitative studies, which examined factors influencing patient/witness response to acute stroke symptoms (*n* = 19 stroke patients, *n* = 26 stroke witnesses) and perceptions about raising stroke awareness in primary care (*n* = 30 clinicians). Both studies included questions about the ‘Act FAST’ campaign. Interviews were content analysed to determine campaign awareness, perceived impact on decisions and response to stroke, and views of the campaign.

**Results:**

Most participants were aware of the Act FAST campaign. Some patients and witnesses reported that the campaign impacted upon their stroke recognition and response, but the majority reported no impact. Clinicians often perceived campaign success in raising stroke awareness, but few thought it would change response behaviours. Some patients and witnesses, and most primary care clinicians expressed positive views towards the campaign. Some more critical participant comments included perceptions of dramatic, irrelevant, and potentially confusing content, such as a prominent ‘fire in the brain’ analogy.

**Conclusions:**

Act FAST has had some perceived impact on stroke recognition and response in some stroke patients and witnesses, but the majority reported no campaign impact. Primary care clinicians were positive about the campaign, and believed it had impacted on stroke awareness and recognition but doubted impact on response behaviour. Potential avenues for optimising and complementing mass media campaigns such as ‘Act FAST’ were identified.

## Background

Mass media campaigns are increasingly used to raise awareness of the signs and symptoms of stroke, and the need to immediately contact emergency medical services (EMS) [1-3]. Stroke awareness campaigns target the general population, including individuals who might experience stroke symptoms and those who might witness stroke. Many campaigns also target primary care clinicians to raise stroke awareness [1].

In England the Department of Health has recently rolled-out the first national stroke awareness raising campaign ‘Act FAST’ [4] between February 2009 and March 2012.^a^ The campaign included television, press and radio advertisements targeting the general population. In addition, awareness raising activities were aimed at primary care clinicians including emails, newsletters, posters and leaflets. Few studies have assessed campaign effects, but these suggest some short-term impact on symptom knowledge and information seeking behaviours [1,5]. However, clear evidence of the effects of the campaign upon response behaviours is currently lacking [6]. These findings have recently been replicated in Ireland which used the ‘Act FAST’ campaign with minor modifications (e.g. Irish voiceover) [7] and are in line with previous evaluations of stroke mass media campaigns reporting variable and modest effects on response behaviour and speed [1].

Limited effects of mass media campaigns for stroke on sustaining public awareness of the need for urgent assessment and emergency response behaviour suggest a need to further optimise and develop intervention components [1]. To inform efforts to improve such campaigns, we undertook a qualitative investigation of the awareness, perceived impact of, and views on, the recent English mass media stroke campaign ‘Act FAST’ in stroke patients, stroke witnesses and primary care clinicians. To date, no research on perceptions of ‘Act FAST’ in different target groups has been conducted. The specific research questions were:

1. Were patients, witnesses and primary care clinicians aware of the ‘Act FAST’ campaign?

2. Did patients, witnesses and primary care clinicians think that the ‘Act FAST’ campaign impacted upon their decisions about, and response behaviours to, stroke symptoms?

3. What were patients’, witnesses’ and primary care clinicians’ views on the ‘Act FAST’ campaign?

## Methods

This was a qualitative study involving individual semi-structured interviews with stroke patients, stroke witness and primary care clinicians. Stroke patients’ and witnesses’ perceptions were examined as they represent key target groups who could have benefited from the ‘Act FAST’ campaign. Primary care clinicians’ perceptions were examined as they represent the health care professionals closest to the community of individuals at risk of stroke and they play a crucial role in terms of raising awareness of stroke. Data analysed in the current manuscript was generated as part of two separate studies, which examined how, why and when EMS are accessed by stroke patients and witnesses (Study 1) [8,9] and the views of primary care clinicians on raising awareness of stroke (Study 2).

Participants in Study 1 were stroke patients and witnesses who were purposively selected from three stroke units in north-east England to include a range of health service contacts and response times. Health service contacts were: telephoning EMS (i.e. dialling 999), telephoning the primary care physician office (i.e. general practice surgery) and presenting to the emergency department (ED). Response times to health service contact included within or after 1 hour following the onset of stroke symptoms. All purposive sampling categories were based on self-report, verified with medical staff in cases of uncertainty. Potential participants were approached by stroke research nurses and the names of interested individuals were passed to JM. It was not possible to ascertain how many people declined to participate. Interviews were conducted ≤14 days of experiencing or witnessing acute stroke and took place whilst ‘Act FAST’ was being actively disseminated (between April 2009 and January 2010). One researcher (JM) conducted all interviews which took place in participants’ homes and were audio-recorded, transcribed and anonymised. Interview questions relevant to the current research were: “*Are you aware of/have you seen the Department of Health’s awareness raising campaign for stroke? If yes, what impact did that have on your decision/actions?*”.

Participants in Study 2 were primary care clinicians who were recruited through the local primary care research network supported by NHS North of Tyne who contacted primary care practices and passed details of interested practices on to JM. Practices were purposively selected with the aim to generate diversity regarding geographical location of general practices (urban vs. rural) and profession of primary care clinicians (general practitioner, practice nurse, health care assistant). All purposive sampling categories were based on geographical information obtained by the researchers and self-report by participants. Altogether 19 practices were invited to participate and 13 accepted. Interviews were conducted between August 2011 and January 2012. Two researchers (JM, SUD) conducted all interviews, which took place within primary care facilities and were audio-recorded, transcribed and anonymised. Interview items relevant to the current research were: “*Are you aware of the Department of Health’s ‘Act FAST’ campaign? What do you think about it?*” and “*Do you think the campaign has impacted on patients’ awareness of stroke symptoms?*”.

All participants provided written, informed consent prior to participation. Studies received ethical approval from the National Health Service (NHS) Sunderland Research Ethics Committee (REC08/H0904/104; REC11/NE/0061).

### Analysis

Interview transcripts were content analysed [10] guided by the three research questions. Codings were made in NVivo9 using a three-step process. First, responses to questions directly assessing views on ‘Act FAST’ were coded. Second, any other unprompted responses regarding ‘Act FAST’ mentioned spontaneously as part of the overall interviews were coded. Third, within codings, responses that related to the three research questions were explored, examining: campaign awareness (defined as a direct encounter of any component of the campaign, e.g. posters or advertisements); views on the campaign (defined as any value judgement expressed in relation to the campaign); perceived campaign impact (defined as judgements of impact on recognition or response to stroke symptoms).

Frequency counts are reported for closed question items that were uniformly prompted across the included sample populations. This included questions regarding campaign awareness (research question 1) and perceived impact on decision/response (research question 2). All codings were made separately for all three participant groups (i.e. patients, witnesses and primary care clinicians).

Throughout the manuscript, quotes for patients, witnesses and primary care clinicians are abbreviated as ‘P’, ‘W’, and ‘PCC’, respectively, followed by the participant number. Patient and witness quotes also include an indication of response behaviour (i.e. telephoning EMS [999], telephoning primary care surgery [GP] and emergency department visit [ED]) and speed of response (i.e. longer [>1 h] or shorter [<1 h] than 1 hour following the onset of stroke symptoms). The specific professional categories are reported for primary care clinicians.

## Results

Nineteen patients (11 women, 8 men), 26 witnesses (21 women, 5 men) and 30 primary care clinicians (24 women, 6 men) were interviewed. The patients were aged 41 to 86 years. Witnesses’ relationships to the patients were: wife/husband (*n* = 14), son/daughter (*n* = 9), nephew (*n* = 1), formal care-giver (*n* = 1) and acquaintance (*n* = 1).

Patient health service contacts within 1 hour of symptom onset were all made to EMS (*n* = 5), whereas those made after 1 hour included primary care surgery (*n* = 8), ED (*n* = 3) and EMS (*n* = 3). Ten patients were able to make health service contacts themselves, the remaining contacts (*n* = 9) were made by other individuals on behalf of the patient.

Most witness contacts with EMS (n = 13) and some to primary care physician offices (n = 2) were made within 1 hour of symptom onset. For those who responded after 1 hour, contact included EMS calls (n = 7), primary care physician (n = 3) and ED visits (n = 1).

Primary care clinicians were recruited from 13 different practices (7 urban, 6 rural) and included 14 general practitioners, 10 practice nurses and 6 health care assistants. All male clinicians were GPs. Job experience ranged from 3 weeks (GP) to 33 years (practice nurse). Ages of witnesses and primary care clinicians were not recorded.

### Stroke patients

#### Awareness of ‘Act FAST’

The majority of stroke patients reported being aware of the campaign overall (*n* = 14/19) at the time of experiencing the stroke. Most recalled having seen the television advertisements (*n* = 11; “*I’ve seen this latest thing on the television where the mouth droops and they can’t smile and the speech is affected*”, P02, GP > 1 h). Other channels through which the campaign reached patients were posters in primary care (*n* = 2; “*It’s up in our doctors on the side of the wall, ring 999 if you’re worried*”, P04, GP > 1 h) or the radio (*n* = 1; “*I had heard about it on the radio*”, P12; 999 > 1 h).

#### Perceived impact on stroke recognition and response behaviour

Two patients reported being influenced by the campaign. One patient described how the campaign helped to recognise symptoms as stroke.

*“Were it not for the benefit of having seen that on the television, it would have taken me an awful lot longer…to realise that [I had a stroke], so I do think that I benefitted a lot from actually seeing those adverts”,* P10, 999 < 1 h

Another patient outlined how the campaign influenced recognition as well as response to stroke symptoms.

*“I knew I was having a stroke […] because of what I’d seen on the television, the way my mouth went. I was drinking coffee and it came all the way out the side […] that’s why I says to [witness]: ‘You’d better call the paramedics’ and then they were here within minutes”*, P11, 999 < 1 h

One patient was unsure whether the campaign had any impact in relation to the stroke episode, but reported an impact on general awareness of health issues.

*“It might have done a little bit but, it just made me more conscious that you’ve got to watch one’s health when you get a bit older”*, P12, 999 > 1 h

The majority of patients who were aware of the Act FAST campaign reported that the campaign had no impact on stroke recognition or response (*n* = 11/14). Some patients commented on the mismatch between the severity of advertised stroke symptoms and the stroke experience.

*“You just think of somebody sitting there and they can’t move their arm. My arm was numb but it wasn’t where I couldn’t move it”*, P05, GP > 1 h

Some patients commented on the campaign’s visual image of a flame within the head as an analogy for stroke, and might have consequently identified the head as the main location for symptoms (*“My head wasn’t bad or anything, it was just my lip and the fingers”*, P17, GP > 1 h). A mismatch between expected and experienced symptoms was reported to prevent the recognition of stroke.

*“It didn’t [fit my experience] because it wasn’t a very bad headache, it was just sort of disorientated at first, you know. And I think you know that’s why I just thought it wasn’t a stroke”*, P04, GP > 1 h

One patient reported relating the television advertisement to other peoples’ past experience rather than the possibility of experiencing a stroke oneself.

*“…when I saw that flame for a start [I thought]: ‘ooh that’s what must have happened to [husband who previously had a stroke]’, I wasn’t thinking about myself”*, P17, GP > 1 h

#### Views on ‘Act FAST’

Patients voiced few views on the campaign. Some stated that they paid little attention to the advertisement (*“I never really paid it that much attention to be quite honest”*, P06, ED > 1 h), with others commenting negatively on the style of the campaign (*“I think is very dramatic”*, P05, GP > 1 h; *“They* [the television advertisements] *were brilliant”*, P10, 999 < 1 h) and length of campaign delivery (*“… [the television advertisements] had been on quite a long time”*, P18, 999 < 1 h).

### Stroke witnesses

#### Awareness of ‘Act FAST’

All witnesses except two (*n* = 24/26) reported having heard of the campaign prior to the time of witnessing the stroke. Out of the witnesses who were aware of the campaign all but one (*n* = 23/24) mentioned having seen the televised advertisement, with the one remaining reporting having seen it on posters in the GP practice (*n* = 1).

#### Perceived impact on stroke recognition and response behaviour

Some witnesses stating campaign awareness (*n* = 9/24) reported having been influenced by it in terms of stroke recognition (*“I had a vague idea of what happened plus the advertisement on the television that I have seen quite a few times and I did think that’s what it was”*, W09, 999 < 1 h) and response to stroke symptoms (*“I think it made me realise that I had to get help quick”*, W17, 999 < 1 h). One witness reported a negative impact of the campaign due to misdiagnosis leading to delay (*“I recognised the TV [advertised symptoms and] said: ‘Can you put your arms up?’ He says: ‘I’m putting my arms up’. I’m thinking: ‘Well it cannot be a stroke’”*, W04, GP > 1 h). Some witnesses reported that the severity and pattern of symptoms advertised by the campaign were not in line with the experienced stroke episode.

*“It wasn’t following the pattern that you see on television advert which you take notice of, it wasn’t following that pattern her speech hadn’t gone, it was delayed, but hadn’t gone and she was still able to move her arms”*, W06, GP > 1 h

Three witnesses stating campaign awareness (*n* = 3/24) reported being unsure whether it impacted on their recognition or response (*“I don’t know if I would have been alert as much of the stroke if I hadn’t have seen the adverts”*, W03, 999 < 1 h) with the remaining witnesses (*n* = 12/24) reporting to not have been influenced in recognition or response by the campaign (*“I don’t think so, I think I would have done it anyway”*, W13, 999 > 1 h).

#### Views on ‘Act FAST’

Several witnesses voiced positive views towards the campaign, including comments on the execution of the television advertisement,as well as the targeting of a broad audience not only including prototypical stroke cases.

*“I think it’s a good advert … puts a point across doesn’t it, you know, like fire we’ve got to get it put out right straight away, that’s it, obviously the subconscious sort of thing like, isn’t it?”*, W10, 999 > 1 h

*“I think it’s good that it isn’t just [focusing on] residential homes, [or] nursing homes so people are targeted - it’s everybody - so even if say* [witnesses’ nephew] *was at home with his grandmother and it happened to her he would know what to do”*, W01, 999 < 1 h

One witness commented positively on the memorability of the television advertisements (“*I don’t usually watch advertisements on the television but that one sort of stuck in my mind*”, P09, 999 < 1 h), whereas another witness reported the opposite effect (“…*it just goes straight over my head to tell you the truth*”, W19, 999 > 1 h). Although the majority of opinions were positive, a few critical comments were voiced during interviews. A further witness found the television advertisements misleading, highlighting that these might create false expectations of stroke

*“It’s a misleading advert. For people who’ve never had […] to deal with strokes before I think that it’s a real bells ringing, you know, shit this is going to happen. That’s very misleading”*, W25, 999 < 1 h

### Primary care clinicians

#### Awareness of ‘Act FAST’

All primary care clinicians were aware of the campaign through campaign engagement with primary care (*“Yes we got the information through the post”*, PCC22, PN), as well as through the media (*“I’ve seen two adverts the one with the male and the one with the female”*, PCC13, GP).

#### Perceived impact on patient recognition and response behaviour

The majority of primary care clinicians (*n* = 13/22) perceived the campaign to have impacted on patient awareness, many basing this judgement on interactions with patients about stroke (*“Yes, it does [raise awareness], because we’ve got patients often … people would often mention it”*, PCC04, GP). The remaining primary care clinicians were either unsure (n = 7/22; *“I hope it has, but I’m not sure”*, PCC05, HCA) or did not perceive the campaign to have made an impact on awareness (*n* = 2/22). One primary care clinician compared ‘Stroke - Act F.A.S.T.’ with another recent campaign that was perceived as prompting more dialogue between patients and health care professionals, which was often seen as a proxy measure of patient awareness.

*“Maybe it wasn’t the right campaign I’m afraid. Because what I do know is at the moment the COPD campaign, I’ve got people mentioning it to me all the time”*, PCC18, GP

Despite many primary care clinicians perceiving increased awareness, only a few (*n* = 3/22) perceived the campaign to have impacted on patient responses to stroke symptoms (*“I think people are more aware and call for help sooner”*, PCC04, GP). The majority (*n* = 14/22) remained unsure of campaign impact on response behaviour (*“I don’t know how much a success it’s seen as”*, PCC01, GP). Some primary care clinicians commented on their inability to judge campaign impact due to the difficulty of detecting a lack of patient contact in case of campaign effect on response behaviour.

*“I suppose in some ways we might not [know if the campaign affects stroke response] because what would happen is they would actually bypass us so we wouldn’t, you know we would only find out when they came out of hospital”*, PCC16, GP

Some primary care clinicians assumed that the campaign impacted on the speed of response, but not on the health service that people contact.

*“It has made a difference to their responses because I think they do phone, they probably do phone more quickly, but they probably still phone us”*, PCC03, GP

One primary care clinician attributed changes in response to stroke to a general trend and noted that many patients still delay and present to the wrong service.

*“I think not necessarily that programme but I think people in general are more keen to present with those symptoms. But I still get people ringing up on a Monday saying their leg went weak on a Saturday, you know, they don’t necessarily present quickly”*, PCC15, GP

Some primary care clinicians (*n* = 5/22) remained unconvinced that the campaign affected patient response behaviours in the event of stroke.

*“Certainly over the last few months the people I have seen who have had those symptoms haven’t changed their behaviour, […] there’s no obvious sign of them going very quickly into 999 rather than coming to see us”*, PCC14, GP

One primary care clinician noted a lack of appropriate response despite correct recognition symptoms as stroke.

*“Some people, yes they know all about it and then you still get them ringing in and saying: ‘I think my mother’s had a stroke’ and you just think ‘Why haven’t you just dialled 999 like they tell you on the television’”*, PCC20, PN

#### Views on ‘Act FAST’

The vast majority of primary care clinicians held positive views about the campaign itself (*“I thought it was good”*, PCC11, GP) and/or the principle of raising awareness (*“Anything that makes people aware is good”*, PCC18, GP). Other adjectives used to describe the campaign were ‘helpful’, ‘informative’, ‘clear’, and ‘powerful’. One primary care clinician was complementary about the ‘fire in the brain’ analogy for stroke.

*“I liked where they described it as a fire in the brain that was quite a visual [image]. Yes you can talk to people about ‘Did you see that advert and the fire destroys your brain and that’s what a stroke would do’, kind of thing”*, PCC20, PN

Primary care clinicians also commented on the campaign’s visibility (*“It’s probably one of the most visual campaigns”*, PCC01, GP) and simplicity (*“They’ve kind of kept it quite simply and compact”*, PCC05, HCA). Some primary care clinicians expressed both critical and positive comments regarding the content (*“It’s alright. It’s like all health campaigns which means that it’s massively over inclusive”*, PCC14, GP) and style of the campaign (*“…possibly over dramatic, but they did kind of bring the point across”*, PCC13, GP). One primary care clinician suggested a frequent change of campaign materials to continue attracting attention.

*“I think it’s like anything else that, if it’s there too often you ignore it, […] you get familiar with the poster that has the FAST on, and so you don’t see it. So I think campaigns need to be changed regularly to then catch somebody’s attention”*, PCC02, PN

Some more critical remarks were voiced regarding the potential impact on individuals (*“Distressing I think for some people”*, PCC21, PN) as well as the limited coverage of all relevant stroke symptoms (*“It only covers some symptoms of stroke, it doesn’t cover everything”*, PCC07, GP).

## Discussion

The majority of patients and witnesses reported campaign awareness at the time of the experienced/observed event, mostly though television advertisements. Additionally, all primary care clinicians reported campaign awareness through both television advertisement and campaign dissemination in primary care. This high level of awareness validates the relevance of participant responses on perceived impact of, and views on, the campaign. A few patients and some witnesses reported campaign impact in terms of decision and response to the stroke situation. Clinicians often perceived the campaign as successful in raising patient awareness of stroke symptoms, but only a few thought it would change response behaviours. Many participants expressed positive views of the campaign, especially clinicians, but some critical comments were voiced, potentially suggesting avenues for optimising stroke mass media campaigns such as ‘Act FAST’.

### Strengths and limitations

This is the first study to qualitatively examine perceived impact and views on the ‘Act FAST’ campaign in key target populations during the time of campaign dissemination. This study thus provides unique insights from selected target populations, which might contribute towards understanding how mass media interventions are perceived and potentially suggesting avenues to inform future campaign optimisation efforts.

Some limitations should be kept in mind when interpreting this research. All data reported in this study is based on retrospective recall, which is prone to cognitive biases and may be inaccurate or incomplete. Second, although patients and witnesses were directly prompted on campaign awareness and perceived impact on decisions/action, no specific prompts on their views on the campaign were included in the topic guide. Consequently, patient and witness views outlined in the paper have been voiced spontaneously and further prompting could have uncovered additional findings. Third, participants in this study were purposively selected samples from relevant key groups to allow conceptual generalisability, limiting the strength of conclusions that can be drawn beyond these populations. Fourth, patient and witness perceptions were assessed after the first and throughout the second active wave of the ‘Act FAST’ campaign and some of the findings might have changed as a result of subsequent phases. Lastly, patients and witnesses were prompted to reflect on campaign impact on decisions/actions. This left participants the freedom to interpret the term “impact” which could be understood as impact on the understanding of stroke, behaviour in relation to the stroke situation, or both.

### Comparison with existing literature and implications

A few patients and some witnesses who stated campaign awareness perceived it to have impacted on stroke recognition and response. Compared to patients, more witnesses perceived an impact on recognition and response, potentially reflecting the observational point of view taken by ‘Act FAST’ (i.e. symptoms were described from an outsider’s perspective, rather than the perspective of the person experiencing the symptoms). Primary care clinicians often perceived the campaign to have impacted on patent/public awareness, but few believed that patient/witness responses were affected in the event of stroke. This perception is in line with the empirical evidence on the campaign, suggesting increases in awareness and minimal short-term behavioural impact [5-7].

Stroke patients and witnesses stating campaign impact reported that the influence of the campaign was decisive in terms of influencing stroke recognition and response behaviours, highlighting the promise of mass media campaigns for stroke. Moreover, participants that reported campaign impact all engaged with health services within 1 hour, with most contacting EMS. However, most participants perceived no impact of the campaign. The limited perceived campaign impact in some individuals could have several potential explanations. First, a mismatch between the severity and pattern of symptoms portrayed within the campaign and experienced/observed stroke symptoms might have led participants to evaluate the campaign as ‘dramatic’, or ‘misleading’. Perceived exaggeration of symptoms displayed as part of the campaign might be particularly prevalent in strokes of mild to moderate severity, where a discrepancy exists between the severity of experienced/observed symptoms and those advertised by a campaign. Moreover, although the campaign explicitly mentions that EMS should be contacted for any single one of the displayed symptoms, all symptoms were illustrated within the same actors at the same time potentially leading some individuals to make the inference that stroke occurs only if several or all symptoms are observed simultaneously. A further explanation for limited perceived impact might be the description of the displayed symptoms using the FAST mnemonic. The FAST mnemonic does not capture all symptoms of stroke [11], and some patients and witnesses referred to the mismatch between type of symptoms portrayed and those experienced or witnessed as a reason for delayed recognition and response. A recent study of recorded emergency calls for stroke showed that, despite the ‘Act FAST’ campaign, less than 5% of callers reported any of the symptoms highlighted through the campaign [12]. The FAST assessment tool was initially developed for use by EMS paramedics [13,14], and not on symptom presentations in patients with acute stroke.

Second, the campaign uses ‘fire in the brain’ as an analogy for stroke occurring. For example, the first line of the televised advertisement states: “*When stroke strikes, it spreads like a fire in the brain, the longer it goes undetected, the more damage is done*” [15]. The intentional use of “hard-hitting imagery” [16] (i.e. a burning head and simulation of stroke symptoms) and threatening metaphors (i.e. comparing stroke with ‘a fire in the brain’) might have reduced acceptability of campaign content and impact for some individuals. Though popular with many primary care clinicians in the current study, strong evidence suggests that using fear appeals within public health campaigns is of limited effectiveness unless combined with high efficacy messages, including the ability to respond (i.e. self-efficacy) and beliefs in the response averting the threat (i.e. response efficacy) [17-19]. However, the ‘Act FAST’ campaign focused mainly on displaying stroke as a threat, rather than efficacy of the desired response behaviour (i.e. calling EMS) and omitted any reference to the effectiveness of thrombolysis, despite this being the underlying drive for the campaign.

Thirdly, the campaign might have omitted relevant evidence and theory in the process of designing campaign content [20]. For instance, the main focus of the campaign seems to be the raising of awareness of stroke symptoms, although a considerable amount of evidence suggests an imperfect relationship between stroke symptom knowledge and reduced delay [21-23]. An emphasis specifically targeted at response behaviour, and a strengthening of the link between stroke symptoms and the need to immediately respond by calling EMS might add further value to stroke mass media campaigns such as ‘Act FAST’. Recent research has shown that increasing explicitness of the required response behaviour together with repeat exposure have had positive impacts on emergency service engagement [24]. In addition, mass media campaign evidence suggests that potential effects on awareness and stroke response are unlikely to be maintained unless supplemented by additional and repeated screening of the campaign [25]. In the case of the ‘Act FAST’ campaign, supplemental access to the message by other means such as during face-to-face consultations with primary care professionals might further consolidate campaign effects. Such supplemental access might provide an opportunity to individually tailor stroke mass media campaign messages to patients, thus potentially overcoming some of the limitations mentioned above.

In conclusion, although views of the first English national awareness raising campaign for stroke were generally positive, patients, witnesses and primary care clinicians often reported limited impact upon an emergency response at the onset of stroke symptoms. Various avenues for optimisation of stroke mass media campaigns such as ‘Act FAST’ were identified: a specific focus on behaviour, repeated campaign exposure, as well as delivering and individually tailoring complementary components through additional channels might further consolidate and increase campaign effectiveness.

## Conclusion

For stroke, there is room for improvement in public awareness of the need for urgent assessment and emergency response. Mass media campaigns for stroke have some impact on awareness and response behaviours, but need to be further optimised for a sustained impact. The recent English stroke awareness raising campaign ‘Act FAST’ has targeted an improvement in stroke awareness and emergency response behaviours. The current research suggests that the ‘Act FAST’ campaign is mainly viewed positively by the key populations of stroke patients, stroke witnesses and primary care clinicians. Despite positive views on the ‘Act FAST’ campaign, perceived impact on individuals’ response to stroke is low. Low perceived impact may relate to specific campaign elements such as symptom display and patterns which point at avenues to inform future campaign optimisation efforts.

## Endnote

^a^To date, Act F.A.S.T. has been rolled out in four active waves: Wave 1 from February to March 2009, Wave 2 from November 2009 to March 2010, Wave 3 in March 2011, and Wave 4 from February to March 2012. All waves included at least television advertisements, with the earlier waves including additional dissemination channels such as radio, the press, and outdoor media.

## Competing interests

The authors declare that they have no competing interest.

## Authors’ contributions

GF, MW, RT, HR, MJM, MPE, FFS, VAS, SUD, and JEM conceived the studies. SUD and JEM collected the data. SUD and JEM analysed the data. SUD wrote the first draft of the manuscript. All authors have read and commented on the manuscript and approved it.

## Pre-publication history

The pre-publication history for this paper can be accessed here:

http://www.biomedcentral.com/1471-2458/13/915/prepub

## References

[B1] Lecouturier J Rodgers H Murtagh MJ White M Ford GA Thomson RG Systematic review of mass media interventions designed to improve public recognition of stroke symptoms, emergency response and early treatment BMC Public Health 2010 10 784 10.1186/1471-2458-10-784 21182777 PMC3022856

[B2] Davis SM Community stroke education using mass media: past results and future implications Stroke 2007 38 7 2034 2035 10.1161/STROKEAHA.107.488312 17540960

[B3] Hodgson C Lindsay P Rubini F Can mass media influence emergency department visits for stroke? Stroke 2007 38 7 2115 2122 10.1161/STROKEAHA.107.484071 17540967

[B4] Stroke: Act F.A.S.T http://www.nhs.uk/Actfast/Pages/stroke.aspx

[B5] National Audit Office Progress in improving stroke care 2010 London: The Stationary Office

[B6] Robinson TG Reid A Haunton VJ Wilson A Naylor AR The face arm speech test: does it encourage rapid recognition of important stroke warning symptoms? Emerg Med J 2013 30 6 467 471 10.1136/emermed-2012-201471 22764171

[B7] Mellon L Hickey A Doyle F Dolan E Williams D Can a media campaign change health service use in a population with stroke symptoms? Examination of the first Irish stroke awareness campaign Emerg Med J 2013 Epub ahead of print 10.1136/emermed-2012-202280 23892414

[B8] Mackintosh JE Murtagh MJ Rodgers H Why People Do, or Do Not, Immediately Contact Emergency Medical Services following the Onset of Acute Stroke: Qualitative Interview Study PLoS One 2012 7 10 10.1371/journal.pone.0046124 PMC3464281 23056247

[B9] Dombrowski SU Sniehotta FF Mackintosh J White M Rodgers H Thomson RG Murtagh MJ Ford GA Eccles MP Araujo-Soares V Witness response at acute onset of stroke: a qualitative theory-guided study PLoS One 2012 7 7 10.1371/journal.pone.0039852 PMC3401234 22911691

[B10] Krippendorff K Content analysis: an introduction to its methodology 2004 Thousand Oaks, CA: Sage

[B11] Bray JE O’Connell B Gilligan A Livingston PM Bladin C Is FAST stroke smart? Do the content and language used in awareness campaigns describe the experience of stroke symptoms? Int J Stroke 2010 5 6 440 446 10.1111/j.1747-4949.2010.00484.x 21050398

[B12] Jones SP Carter B Ford GA Gibson JME Leathley MJ McAdam JJ O’Donnell M Punekar S Quinn T Watkins CL The identification of acute stroke: an analysis of emergency calls Int J Stroke 2013 8 408 412 10.1111/j.1747-4949.2011.00749.x 22335960

[B13] Harbison J Hossain O Jenkinson D Davis J Louw SJ Ford GA Diagnostic accuracy of stroke referrals from primary care, emergency room physicians, and ambulance staff using the face arm speech test Stroke 2003 34 1 71 76 10.1161/01.STR.0000044170.46643.5E 12511753

[B14] Harbison J Massey A Barnett L Hodge D Ford GA Rapid ambulance protocol for acute stroke Lancet 1999 353 9168 1935 10.1016/S0140-6736(99)00966-6 10371574

[B15] Know the signs of stroke http://www.nhs.uk/actfast/Pages/know-the-signs.aspx

[B16] Stroke http://webarchive.nationalarchives.gov.uk/+/www.dh.gov.uk/en/Healthcare/Longtermconditions/Vascular/Stroke/index.htm

[B17] Witte K Allen M A meta-analysis of fear appeals: implications for effective public health campaigns Health Educ Behav 2000 27 5 591 615 10.1177/109019810002700506 11009129

[B18] Ruiter RAC Abraham C Kok G Scary warnings and rational precautions: a review of the psychology of fear appeals Psychol Health 2001 16 6 613 630 10.1080/08870440108405863

[B19] Peters GJY Ruiter RAC Kok G Threatening communication: a critical re-analysis and a revised meta-analytic test of fear appeal theory Health Psychology Review 2013 7 1 S8 S31 23772231 10.1080/17437199.2012.703527 PMC3678850

[B20] Noar SM A 10-year retrospective of research in health mass media campaigns: where do we go from here? J Health Commun 2006 11 1 21 42 10.1080/10810730500461059 16546917

[B21] Teuschl Y Brainin M Stroke education: discrepancies among factors influencing prehospital delay and stroke knowledge Int J Stroke 2010 5 3 187 208 10.1111/j.1747-4949.2010.00428.x 20536616

[B22] Jones SP Jenkinson AJ Leathley MJ Watkins CL Stroke knowledge and awareness: an integrative review of the evidence Age Ageing 2010 39 1 11 22 10.1093/ageing/afp196 19897540

[B23] Lecouturier J Murtagh MJ Thomson RG Ford GA White M Eccles M Rodgers H Response to symptoms of stroke in the UK: a systematic review BMC Health Serv Res 2010 10 157 10.1186/1472-6963-10-157 20529351 PMC2911429

[B24] Bray JE Mosley I Bailey M Barger B Bladin C Stroke public pwareness pampaigns pave increased ambulance dispatches for stroke in Melbourne, Australia Stroke 2011 42 2154 2157 10.1161/STROKEAHA.110.612036 21757668

[B25] Wakefield MA Loken B Hornik RC Use of mass media campaigns to change health behaviour Lancet 2010 376 9748 1261 1271 10.1016/S0140-6736(10)60809-4 20933263 PMC4248563

